# Metatranscriptomics profile of the gill microbial community during *Bathymodiolus azoricus* aquarium acclimatization at atmospheric pressure

**DOI:** 10.3934/microbiol.2018.2.240

**Published:** 2018-03-20

**Authors:** Inês Barros, Hugo Froufe, George Marnellos, Conceição Egas, Jennifer Delaney, Michele Clamp, Ricardo Serrão Santos, Raul Bettencourt

**Affiliations:** 1 Department of Oceanography and Fisheries, University of the Azores, 9901-862 Horta, Portugal; 2 MARE-Marine and Environmental Sciences Centre, 9901-862 Horta, Portugal; 3 Next Generation Sequencing Unit-BIOCANT; Parque Tecnológico de Cantanhede, Núcleo 04, Lote 8, 3060-197 Cantanhede, Portugal; 4 Harvard University, Informatics and Scientific Applications, 38 Oxford Street, Cambridge, MA 02138-2020, United States; 5 Harvard University, Biological Laboratories, Room 3085, 16 Divinity Avenue, Cambridge, MA 02138-2020, United States; 6 OKEANOS Center, Faculty of Science and Technology, University of the Azores, 9901-862 Horta, Portugal

**Keywords:** *Bathymodiolus azoricus*, long term acclimatization, metatranscriptome, RNA-seq, host-microbe interactions, symbionts, hydrothermal vent

## Abstract

**Background:**

The deep-sea mussels *Bathymodiolus azoricus* (Bivalvia: Mytilidae) are the dominant macrofauna subsisting at the hydrothermal vents site Menez Gwen in the Mid-Atlantic Ridge (MAR). Their adaptive success in such challenging environments is largely due to their gill symbiotic association with chemosynthetic bacteria. We examined the response of vent mussels as they adapt to sea-level environmental conditions, through an assessment of the relative abundance of host-symbiont related RNA transcripts to better understand how the gill microbiome may drive host-symbiont interactions in vent mussels during hypothetical venting inactivity.

**Results:**

The metatranscriptome of *B. azoricus* was sequenced from gill tissues sampled at different time-points during a five-week acclimatization experiment, using Next-Generation-Sequencing. After Illumina sequencing, a total of 181,985,262 paired-end reads of 150 bp were generated with an average of 16,544,115 read per sample. Metatranscriptome analysis confirmed that experimental acclimatization in aquaria accounted for global gill transcript variation. Additionally, the analysis of 16S and 18S rRNA sequences data allowed for a comprehensive characterization of host-symbiont interactions, which included the gradual loss of gill endosymbionts and signaling pathways, associated with stress responses and energy metabolism, under experimental acclimatization. Dominant active transcripts were assigned to the following KEGG categories: “Ribosome”, “Oxidative phosphorylation” and “Chaperones and folding catalysts” suggesting specific metabolic responses to physiological adaptations in aquarium environment.

**Conclusions:**

Gill metagenomics analyses highlighted microbial diversity shifts and a clear pattern of varying mRNA transcript abundancies and expression during acclimatization to aquarium conditions which indicate change in bacterial community activity. This approach holds potential for the discovery of new host-symbiont associations, evidencing new functional transcripts and a clearer picture of methane metabolism during loss of endosymbionts. Towards the end of acclimatization, we observed trends in three major functional subsystems, as evidenced by an increment of transcripts related to genetic information processes; the decrease of chaperone and folding catalysts and oxidative phosphorylation transcripts; but no change in transcripts of gluconeogenesis and co-factors-vitamins.

## 1. Introduction

Deep-sea hydrothermal vents are regarded as extreme environments. Despite their challenging physicochemical environmental conditions, life is supported by chemosynthetic systems where highly specialized vent organisms have emerged throughout evolution [1]. *Bathymodiolus azoricus* mussels have adapted well to deep-sea extreme environments and represent the dominating faunal component from hydrothermal vent sites of the Mid-Atlantic Ridge. They owe their successful adaptation and high biomass to specialized exploitation of methane and sulfide sources resulting from venting activity, from their gill endosymbiont bacteria [2],[3]. Their extraordinary capabilities to adapt and thrive in chemosynthesis-based environments, largely devoid of photosynthetic primary production and characterized by rapid geochemical regimes changes, is due to their symbiotic association with chemosynthetic bacteria within their gills [2],[3]. Mutualistic associations between bacteria and eukaryotes occur ubiquitously in nature, forming the basis for key ecological and evolutionary innovations. Symbiotic associations enable marine invertebrate hosts to colonize sulfide and methane rich environments, in which the organic matter produced by the sulfur-oxidizing (SOX) and methane-oxidizing (MOX) endosymbionts ensures the nutrition of the host [4],[5]. Chemosynthetic symbioses within Bivalvia are excellent model systems for studying the evolution of bacteria-eukaryote interactions, as they display a range of intricacies with some symbionts housed intracellularly within specialized gill cells called bacteriocytes [6]. Furthermore, the gill tissue represents an interface between the external milieu and the animal's body cavity and acts as an immunity-mediating tissue where circulating hemocytes contribute to the overall immune defense reactions [7].

*B. azoricus* has been the subject of several studies in our laboratory, aiming at the elucidation of molecular mechanisms underlying holobiont interactions [5],[8]. We have previously shown that the bacterial community structure deduced from 16S rRNA sequencing analyses pointed to an unanticipated similarity of endosymbiont bacterial prevalence between Menez Gwen and Lucky Strike *B. azoricus* gills [8]. Furthermore, the gill tissues have been the focal point of our transcriptomics studies, using high-throughput sequencing technology with the aim of discovering new genes related to deep-sea vent adaptation and immune function, including genes of bacterial origin. As a result, we significantly increased the number of *B. azoricus* genes in the public domain [9].

While different types of microorganisms may form complex systems to undergo biogeochemical processes, the function of microbial communities and their possible relationships with macrofauna dwelling around hydrothermal vents remains largely unknown. In particular, questions regarding how symbionts or microbiomes affect host physiology and which are the mechanisms of adaptation in response to factors external to symbiosis, such as hydrothermal venting heterogeneity, disruption and ultimately venting cessation, remain answered. Such environmental changes likely affect host-symbiont associations that drive key physiological functions probably affecting host fitness and how environmental microbes interact with vent animals.

High-throughput sequencing is now enabling the use of metatranscriptomics strategies where total RNA from prokaryotic-eukaryotic functional consortia is sequenced, revealing active bacterial community members and metabolic pathways [10]. In addition to gene expression investigations providing insights into responses to varying environmental conditions, the dominance of rRNA in metatranscriptomic samples allows the assessment of the entire microbiome, without prior selection of taxonomic groups for this study. This is technically much less challenging than enrichment of mRNA, avoids PCR bias and can be carried out straightforwardly on multiple samples [11].

Furthermore, sequencing the expressed mRNA and rRNA enables the analysis of metatranscriptional profiles, thus revealing previously unknown transcripts from natural communities in response to varying environmental conditions and providing a molecular framework for interpreting metabolic functions in chemosynthetic symbiont associations with deep-sea hydrothermal vent mussels [12],[13].

Currently, various marine bivalve transcriptomes have been sequenced and analyzed, highlighting the expansion of many immune-related gene families in most bivalve taxa examined thus far, probably as a result of adaptation to life in microbe-rich environments [9],[14]. The interdependence between environmental microorganisms and hydrothermal vent mussels may result in long term associations at hydrothermal vent sites, driving host gene expression. These associations may be compromised by the cessation of hydrothermal activity or during acclimatization in an aquarium, without methane and sulfur supplement. This study addresses the question of how gill microbial consortia contribute to the transcriptional status of the vent mussel *Bathymodiolus azoricus*, using a metatranscriptomic approach to examine primarily 16S and 18S rRNA gene phylogenies coupled with the assessment of functional genes, as indicators of microbial and vent mussel activity. In the context of long term acclimatization effects on host reactions relative to the prevalence of endosymbiont bacteria, we conducted a time series study over 5 weeks of acclimatization in plain sea water aquarium conditions and without adding metals, gas or food sources, in a way evoking a hydrothermal vent shut-off episode and subsequent effects on vent animal-microbial associations. The metatranscriptomic analysis of rRNA and mRNA used to profile the gill microbial community and host transcriptional status was achieved through the implementation of a custom bioinformatics pipeline substantially improving taxonomy and functional assignments. Both taxonomy and function showed distinct patterns of variation throughout acclimatization, and provided evidence of dynamical mussel-endosymbiont interaction shifts that led to progressive adaptation to aquarium environment of *B. azoricus*.

## 2. Materials and methods

### 2.1. Ethics statement

No specific permissions were required for the field studies described. *Bathymodiolus azoricus* is not an endangered or protected species.

### 2.2. Specimen collection

*B. azoricus* mussels were collected during the MoMARSAT cruise (July 2011) from the Mid-Atlantic Ridge (MAR) hydrothermal vent field Menez Gwen (850 depth, 37°50′8″–37°51′6″ N; 31°30′–31°31′8″ W), with the French R/V *Pourquoi Pas?* using the Remote Operated Vehicle, Victor 6000.

### 2.3. Experimental apparatus and design

Mussels averaging between 5 and 8 cm were acclimatized in the LabHorta laboratory system using aquaria filled with 20 L of fresh cooled sea water (7–8 °C), not differing much from sampling site, directly pumped from a local coastal area, continuously treated with UV light and filtered through an external power canister filter (Eheim 600), circulating through the aquaria system and without adding metals, gas or food sources. The air-oxygen supply provided oxygen saturation around 10–50% under atmospheric pressure. Experiments were performed in the dark except during water monitoring and sampling. Eight animals, evenly distributed according to size and across 11 time points, were sampled at: 0 hours (0 h), 12 hours (12 h), 24 hours (24 h), 36 hours (36 h), 48 hours (48 h), 72 hours (72 h), 1 week (1 w), 2 weeks (2 w), 3 weeks (3 w), 4 weeks (4 w) and 5 weeks (5 w) of acclimatization, upon which the gills were dissected, flash frozen in liquid nitrogen and stored at −80 °C for subsequent RNA extraction in the laboratory. The 0 h time point sampling was considered as the beginning of the acclimatization experiment, when animals were brought onboard, after deep-sea retrieval and immediately preserved. All subsequent time points were sampled in the LabHorta aquarium laboratory, in Horta, Faial Island, Azores.

### 2.4. RNA extraction

Total RNA from *B. azoricus* gill tissues samples was extracted with TriReagent® (Ambion®/Life Technologies; Carlsbad, CA) and further purified with the RiboPure® Kit (Ambion®/Life Technologies) following the manufacturer's specifications and re-suspended in nuclease-free DEPC-treated water. Total RNA quality preparations and concentrations were assessed by the A260/280 and A260/230 spectrophotometric ratios using the NanoVue spectrophotometer (GE, Healthcare Life Sciences) and Qubit® Fluorometer quantitation (Life Technologies). Total RNA extractions were performed on gill tissues from eight animals for each acclimatization time point. RNA purifications were pooled to generate one RNA extract for each time point tested.

### 2.5. cDNA synthesis and DNA shearing

In collaboration with Prof. Peter Girguis's Laboratory (Department of Organismic and Evolutionary Biology, Harvard University), the total RNA from gill samples was DNAse-digested using the TURBO DNA-free™ Kit (Ambion® Life Technologies). Reverse-transcription was performed using the SuperScript™ III First-Strand Synthesis System (Invitrogen™, ThermoFisher Scientific) with random hexamers priming for first-strand synthesis (50 ng/µl, provided in the SuperScript™ III First-Strand Synthesis System), followed by second-strand synthesis using the SuperScript Double-Stranded cDNA Synthesis Kit for (Invitrogen™ ThermoFisher Scientific). cDNA was purified with the QIAquick PCR Purification Kit (Qiagen; Valencia, CA) and quantified using the Qubit® Fluorometer (Life Technologies). The samples were then sheared into approximately 400 bp fragments using the Covaris S220 ultrasonicator (Covaris; Woburn, MA), with the following parameters: duty cycle, 10%; intensity, 4; 200 cycles per burst; 72 sec. Sheared DNA fragments were concentrated using the DNA Clean & Concentrator™-25 (Zymo Research; Irvine, CA) and quantified using Qubit® Fluorometer (Life Technologies).

### 2.6. RNA-seq library preparation and quality control

DNA libraries were prepared using the Apollo 324™ System with the PrepX™ ILM DNA Library Kit (IntegenX; Pleasanton, CA) according to the manufacturer's instructions. A unique NEXTflex™ DNA indexed adapter was used for each sample (Bioo Scientific; Austin, TX). Libraries were quantified using the Qubit® Fluorometer (Life Technologies) and then amplified according to the Bioo Scientific protocol (10 cycles, using NEBNext^®^ High-Fidelity 2× PCR Master Mix (New England BioLabs^®^; Ipswich, MA)). Cleanup of amplified libraries was performed using a preconfigured workflow on the Apollo 324™ System (IntegenX). Library concentration and size were then verified using the Bioanalyzer 2100 System and High Sensitivity DNA Kit (Agilent Technologies; Santa Clara, CA). Library fragments containing the Illumina adapter sequence were quantified using the KAPA Library Quantification Kit (Kapa Biosystems; Wilmington, MA) according to the manufacturer's instructions.

### 2.7. Library sequencing

The libraries were pooled in equimolar amounts, and a final quantification was achieved with the Qubit®, Bioanalyzer, and a KAPA qPCR assay (final concentration 12.3 nM and fragment size around 428 bp). A 150 bp paired-end Rapid Run was performed on a HiSeq 2500 (Illumina, Inc.; San Diego, CA). Sequencing was carried out at the FAS Center for Systems Biology, Harvard University. All the raw reads were submitted to the Sequence Read Archive (SRA) submission portal (https://submit.ncbi.nlm.nih.gov/subs/sra/) and made available under the SRA accession: SRP126879.

### 2.8. Bioinformatics analysis

The fastq files with the reads of the 11 sequenced samples were processed for trimming routine using the stand alone trimming tool Cutadapt [15] to remove Illumina adapter sequences; the bases with quality below 15; and reads shorter than 25 bp. Sequences were further filtered with PRINSEQ, for removal of duplicate reads and trimming of PolyA tails larger than 5 nucleotides [16]. Subsequently, LSU rRNA, SSU rRNA, and putative mRNA detection was achieved with SortMeRNA software [17] enabling the detection of paired rRNA reads by searching against different rRNA sequence databases. The presence of LSU rRNA was determined by filtering against the default databases that included 23S, 5.8S, 5S, and 28S rRNAs. SortMeRNA “paired-out” option was used to ensure that both reads in a pair were aligned to LSU rRNA. Reads mapped to these database sequences were removed from further analysis. The remaining reads were then evaluated for the presence of SSU rRNA against 16S and 18S rRNA default databases, again with the “paired-out” option. Reads mapping to those databases sequences were classified as SSU read and will be used for OTU analysis. The remaining reads were classified as putative mRNA and further used for function analysis.

To identify the biodiversity in each sample the SSU reads were mapped against the SILVA database version 111 using SortMeRNA tool for filtering, mapping and OUT picking NGS reads in metatranscriptomic and metagenomic data. If both reads in a pair aligned against the same rRNA sequence with 97% identity and 97% query coverage, then the accession number of the rRNA sequence hit was assigned to the paired read. Paired reads that did not meet these criteria were discarded. Finally, a cluster map file was created based on the accession number to be used in QIIME [18]. These two steps were performed by an in house script. The cluster map was then used to create an OTU table in BIOM format using the QIIME script *make_otu_table.py* and a SILVA taxonomy map file to direct OTU taxonomy attribution using id from SILVA database. The OTU table was summarized using *summarize_taxa_through_plots.py* (QIIME). For testing differences in the microbial community richness between samples, alpha diversity measures (Observed species, Chao1, Shannon index, equitability) were calculated using *alpha_diversity.py* script (QIIME) and Chao1 rarefraction curves using *alpha_rarefaction.py* (QIIME).

To access protein function, putative mRNA reads were mapped against IMG/QIIME reference protein sequences using *map_reads_to_reference.py* (QIIME) with BLAST as the alignment method and default parameters.

**Figure 1.**
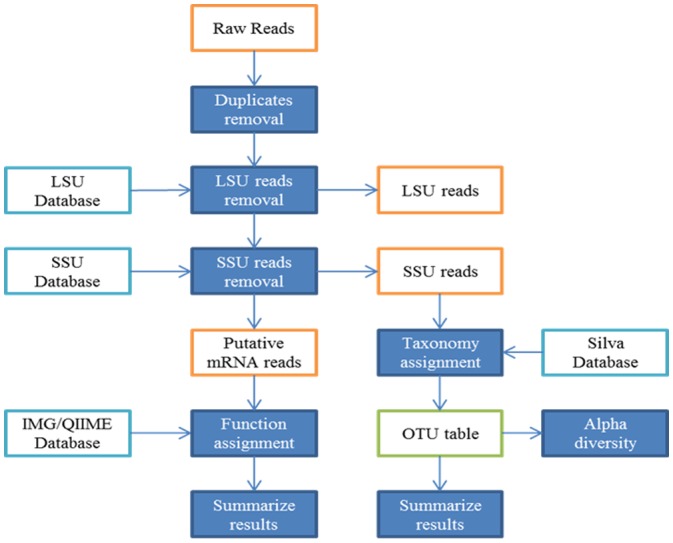
Workflow diagram representing data processing pipeline for metatranscriptome analysis and annotation of *B. azoricus* gill holobiome.

Because *map_reads_to_reference.py* could not process both reads in a pair simultaneously, putative mRNA read pairs were divided, and each read was processed independently, producing two sets of results from each sample, one for each pair read. This output was further processed with *summarize_taxa_through_plots.py* to produce final plots. The outline of the workflow is illustrated in Figure 1.

## 3. Results and discussion

Natural interactions between environmental microorganisms and vent mussels may support long term associations driving host gene expression which, in the event of venting activity cessation, may undergo transcriptional shifts and metabolic activity changes. Assessing the microbial diversity in *B. azoricus* gill tissues will likely bring insight into how bacteria community structures are affected by acclimatization processes at atmospheric pressure in aquaria conditions and how bacterial gene expression is influenced by host physiological status under experimental challenges. With our RNA-seq approach we aimed to study organism diversity present in *B. azoricus* gills as well as genes expressed in *B. azoricus* and in the bacterial community.

A comprehensive transcriptome data set from *B. azoricus* gill tissues corresponding to 11 different acclimatization time-points was obtained with the HiSeq Illumina sequencing platform; it consisted of a total of 181,985,262 150 bp paired-end reads, with an average of 16,544,115 paired-end reads per sample (Table 1). On average, samples contained 42.41% unique read being these, 26.73% LSU rRNA, 11.27% SSU rRNA and 4.42% putative mRNA. Taking into consideration the presence of *B. azoricus* gill endosymbionts [5], we enriched bacterial cDNAs by performing reverse transcription with random hexamers without oligo-dT. Poly-A mRNA enrichment or rRNA depletion was not performed because this procedure would have removed bacterial mRNA and all rRNA, leaving only mRNA from *B. azoricus*. rRNA depletion decrease the total number of rRNA sequences while compromising organism detection based on 16S and 18S rRNA genes. This explains why we obtained a high percentage of rRNA sequences. Overall, this sequencing analysis approach, using both rRNA and mRNA transcripts, allowed direct access to the prokaryote and eukaryote biodiversity of *B. azoricus* gills acclimatized in aquarium conditions at atmospheric pressure.

**Figure 2.**
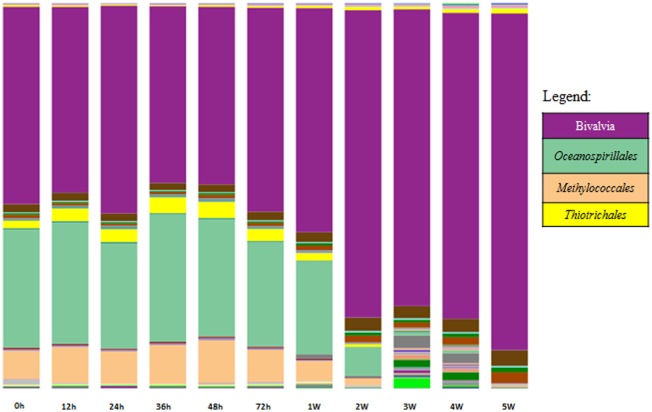
Taxonomy summary plots. 16S and 18S OTUs taxonomical assignments are shown as a bar chart. Abundance of 16S and 18S reads assigned to each taxon reflects the height of the bar. Main Gammaproteobacteria representatives are shown as *Oceanospirillales* *Thiotrichales* and *Methylococcales*.

**Figure 3.**
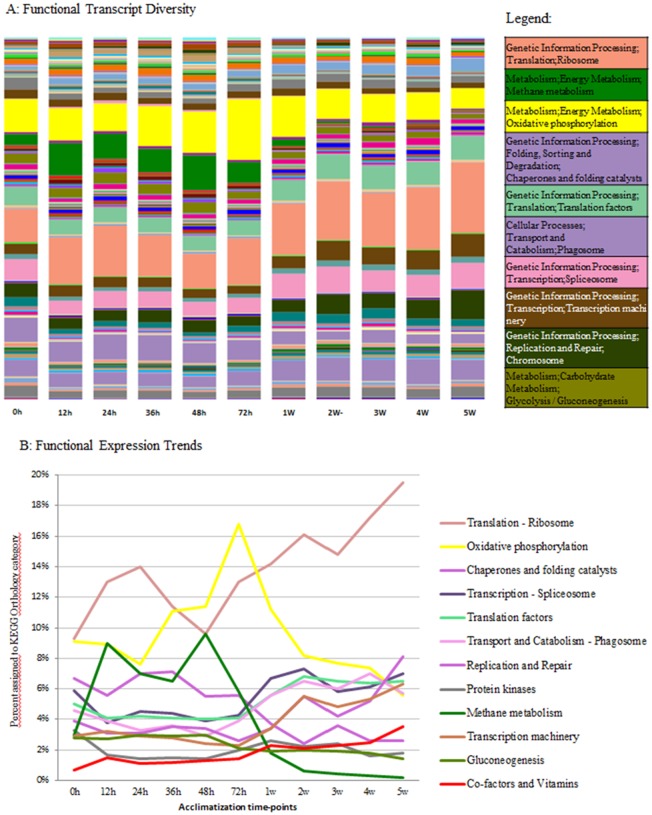
Functional summary plots. A: Functionally important transcript categories of *B. azoricus*-endosymbiont transcriptome according to KEGG database assignment. B: Time-dependent transcript expression trends during experimental acclimatization at atmospheric pressure.

A broad analysis of *B. azoricus* gill microbiome structure revealed specific bacterial transcripts variation across different acclimatization time points, which could mean, on the one hand, changes in bacterial activity linked to the transcriptional activity of the entire gill-microbial community and on the other hand changes of bacterial composition in the samples. Gene expression levels across acclimatization time revealed a pattern of activity consistent with progressive loss of endosymbionts activity and specific metabolic responses to physiological adaptations in aquarium environment (Figures 2 and 3).

### 3.1. Microbial transcripts during acclimatization

Sequencing the small subunit rRNA gene (16S rRNA gene in Bacteria and Archaea or 18S rRNA gene in Eukarya) is a widely applied approach to study the composition, structure and spatiotemporal patterns of microbial communities, due to its ubiquity across all domains of life [19]. The 16S rRNA gene was selected for this study because of its taxonomic resolution, conserved flanking regions, and length. During acclimatization the number of assigned prokaryotic OTUs based on sequenced transcripts, decreased slightly between 0 h to 24 h followed by an increase between 36 h and 4 weeks and finally declining again towards 5 weeks acclimatization time (Table 2). This variation in species diversity is probably reflecting a fluctuating bacterial community differentiating as a result of acclimatization to aquarium environment. The Shannon index decrease pointed at a reduction of bacterial species during acclimatization (Table 2). The Shannon's index accounts for both abundance and evenness of the species which in the present case the index decreases as both richness and the evenness of the community decreases. This is illustrated in Figure 2 showing the vent-associated bacterial community (*Oceanospirillales*, Methylococcales and Thiotrichales) is decreasing during acclimatization mainly after 1 week time point. Moreover, the number of assigned prokaryotic OTUs based on sequenced transcripts increased to higher levels at 3 weeks and 4 weeks's time point, after which time, the original bacterial community composition shifted, and decreased to lowest levels of diversity toward the last time point (Figure 2). Furthermore, the low and decreasing values of equitability through all acclimatization time points also suggest a low diversity dominated by the original vent-associated bacterial community and by Bivalvia sequence assignments during the first week in aquaria. From week 2 toward the end of the acclimatization experiment, a major microbial composition shift was noted characterized by the absence of vent-associated bacteria and an increase of both bacterial diversity and eukaryote species corresponding to Bivalvia and Bryozoa which represented the majority of all taxonomy assignments by the end of acclimatization.

**Table 1.** HiSeq Illumina data set from *B. azoricus* gill tissues.

Time points	Raw data	Duplicates	Dup%	rRNA LSU	rRNA LSU%	rRNA SSU	rRNA SSU%	Putative mRNA	Putative mRNA%	mRNA pair1/pair2
0 h	16 277 124	8 964 678	55.08%	4 159 716	25.56%	1 803 216	11.08%	1 349 514	8.29%	25784/26323
12 h	16 799 796	8 753 076	52.1%	4 804 896	28.6%	2 258 626	13.44%	983 198	5.85%	17029/17580
24 h	15 582 702	8 678 798	55.7%	4 204 320	26.98%	2 120 264	13.61%	579 320	3.72%	18511/19512
36 h	18 844 606	9 950 220	52.8%	5 472 662	29.04%	2 454 942	13.03%	966 782	5.13%	18511/19512
48 h	16 505 724	9 049 980	54.83%	4 714 292	28.56%	2 111 882	12.79%	629 570	3.81%	8395/9076
72 h	18 342 152	9 779 684	53.32%	5 276 664	28.77%	2 185 178	11.91%	1 100 626	6,00%	14898/15301
1 w	16 086 918	9 229 370	57.37%	4 265 494	26.52%	1 791 380	11.14%	800 674	4.98%	16671/16827
2 w	11 755 104	7 012 148	59.65%	3 197 050	27.2%	1 157 994	9.85%	387 912	3.3%	5079/5555
3 w	17 237 480	10 939 364	63.46%	4 232 038	24.55%	1 623 300	9.42%	442 778	2.57%	8959/9350
4 w	18 929 698	12 350 284	65.24%	4 469 100	23.61%	1 653 418	8.73%	456 896	2.41%	8026/8486
5 w	15 623 958	9 989 130	63.93%	3 848 086	24.63%	1 394 758	8.93%	391 984	2.51%	5694/6312

**Table 2.** Alpha diversity estimators from 16S rRNA and 18S rRNA.

Time points	Observed species	Chao1	Shannon index	Equitability	Good's coverage
0 h	1003	1593.5	4.209	0.4222	0.9995
12 h	894	1565.1	4.135	0.4218	0.9996
24 h	793	1352.6	3.970	0.4122	0.9997
36 h	842	1439.4	4.113	0.4233	0.9997
48 h	1060	2027.4	4.300	0.4279	0.9995
72 h	1138	1993.8	4.143	0.4081	0.9995
1 w	1727	2786.9	4.219	0.3923	0.9993
2 w	1199	2103.1	3.415	0.3339	0.9991
3 w	2096	3029.8	3.995	0.3621	0.9991
4 w	2320	3789.1	3.874	0.3465	0.9990
5 w	1342	2298.2	3.045	0.2931	0.9992

**Figure 4.**
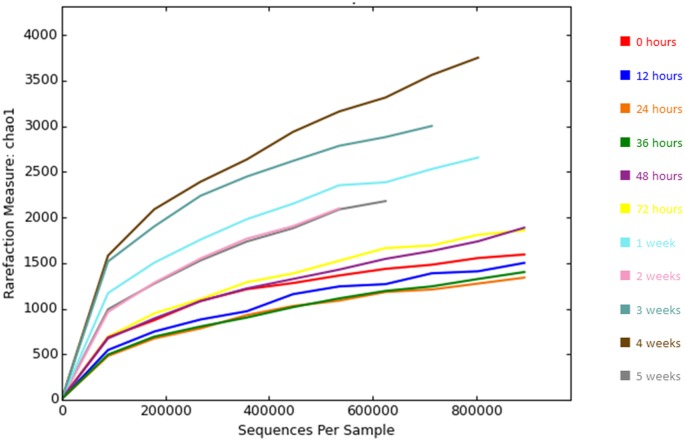
Rarefaction curves of Chao1 diversity.

In addition, the high values of Good's coverage and Chao1 rarefaction curves reached a plateau phase for most of the samples showing that sequencing reads were well covered (Figure 4).

The 16S rRNA gene sequences revealed that species affiliated to Gammaproteobacteria were the dominant active population, until 2 weeks acclimatization. The Gammaproteobacteria class includes representatives of the Thiotrichales and Methylococcales families (Figure 2) which in the case of deep-sea hydrothermal vents are likely chemolithoautotrophic bacteria living in microbial mats from hydrothermal vent fields or surrounding mussel beds. In deep-water habitats the Oceanospirillales phylotype has been found in association with hydrothermal vent and hydrocarbon seep bivalves [20],[21], *Oceanospirillales* comprise marine bacteria; they are widespread aerobic degraders of complex organic compounds, through the excretion of hydrolytic enzymes and emulsifying agents [22]. In the gills of other investigated bivalves, *Oceanospirillales* occured together with methane- and sulfide-utilizing symbionts and can exert direct organism impact through parasitism, symbiosis and algicidal activity [23]. The functions of host-associated *Oceanospirillales* range from parasitic consumption of host tissue to beneficial symbiont interactions that assist in the metabolism or cycling of organic compounds [20],[21].

The 16S rRNA gene sequences that affiliated to *Oceanospirillales*, Methylococcales and Thiotrichales (Figure 2) bring evidence supporting an active bacterial community surrounding the gill tissue with potential methane and sulfur oxidizing activity. The results also provide evidence of *Methylococcales* transcripts in *B. azoricus* gills, likely reflecting the higher concentrations of abiotic methane generated at hydrothermal vent systems. Members of the order Thiotrichales (Gammaproteobacteria) are known for their ability to form biofilms on oxic/anoxic interfaces, using dissolved free oxygen to oxidize reduced sulfur compounds [23].

During the first acclimatization week the relative read abundance was 30% *Oceanospirillales*, Methylococcales 8% and Thiotrichales 3%, after which time a remarkable decrease was seen at 2 weeks acclimatization. Sequences affiliated to *Methylococcales* was interpreted as indicative of metabolic activity during the first 2 weeks of acclimatization supported by methane-oxidizing metabolism; related to gill-endosymbiont, when soluble or membrane bound methane monooxygenase (MMO) is probably still functional. The results are in agreement with our previous studies based on differential expression analyses of functional genes, revealing a diverse and active chemolithoautotrophic population in *B. azoricus* gills [24]. Moreover, our results corroborate previously identified bacterial sequences associated with Gammaproteobacteria, a class of bacteria that includes also hydrothermal vent animal symbionts and representatives of the *Thiotrichales* and *Methylococcales* orders [23]. Interestingly, Beinart et al identified a novel proteobacterial order Oceanospirillales member that that inhabits the gills of the hydrothermal vent snail *Alviniconcha* together with chemosynthetic [22].

Taken together, our results support previous studies estimating higher numbers of methanotrophs in vent associated microbial communities and not necessarily present intracellulary in MAR mussel populations. This is in agreement with higher methane concentrations at the Menez Gwen hydrothermal vent field [24]. However, toward the end of acclimatization to aquarium environment, and in the absence of methane and sulfur supplement, a microbial diversity shift was seen, characterized by the occurrence of opportunistic bacteria identified as *Vibrionales*, *Chromatiales*, *Alteromonadales*, *Campylobacterales*, *Fusobacteriaceae*, *Flavobacteriales*, *Bacteriales* and *Halobacteriales*.

A corresponding eukaryote taxonomy assignment based on 18S rRNA expression profile was observed during the first week of acclimatization (average 50%), increasing significantly after 2 weeks (79.8%), as shown in Figure 2 (purple color).

### 3.2. Transcripts involved in endosymbiosis

#### 3.2.1. Active methane oxidizers

The endosymbiont metabolic pathway was evidenced by the expression levels of the methane monooxigenase (KEGG function K08684) as an indicator of methanotrophy, as shown in Figure 3A (green color). Methane monooxygenase (MMO) transcripts were expressed during the first 72 h and until the first week of acclimatization, after which a sharp decline followed, during the remaining adaptation process in aquaria, leading to a complete absence after 2 weeks. The gradual decrease in the expression of genes from the methane-oxidizing symbiont may correspond to the decrease in symbiont abundance, otherwise if experimentally demonstrated, in this study, could suggest interdependence between methane metabolism and endosymbiont densities over acclimatization time. Indeed, relative symbiont abundances may alter rapidly in response to changes in the availability of their respective substrates [25]. These results confirm our previous experiments using mussels maintained under the same experimental conditions showing that endosymbionts are gradually disappearing from gill tissues, whether through shedding or intracellular digestion as a result of acclimatization at ambient pressure [24],[26].

#### 3.2.2. Active sulfur oxidizers

Surprisingly, while the largest proportion of retrieved prokaryote sequences were assigned to methane-oxidizing groups, only a much smaller fraction of sulfur-oxidizing species was part of the endosymbiotic communities, even at the beginning of the acclimatization experiment (less than 0.5% of the OTUs assignments). Less than 0.5% of the OTU assignment may not capture the true abundance of sulfur-oxidizing bacteria. This may be explained by a lack of sulfur resources available, whether from the natural environment at the time of mussel collection or from low levels of internal storage of partially oxidized sulfur precipitates, which, otherwise, would serve as an alternative energy source for endosymbiont bacterial metabolism requirements and demands for energy, as described previously [24],[27]. Alternatively, the low sequences assignment to sulfur-oxidizer bacteria may suggest possible biases in RNA extraction efficiency, low 16S rRNA gene copy number or sequencing issues.

### 3.3. Energy metabolism transcripts

Symbioses between marine invertebrates and methanotrophs provide bacteria with access to methane and oxygen and other substrates necessary for metabolism and the invertebrate host with a source of organic carbon. Energy metabolism transcripts were categorized as follows according to their representation across the 11 different time points of acclimatization.

#### 3.3.1. Oxidative phosphorylation

Methanotrophic bacteria utilize methane for generating ATP through oxidative phosphorylation and for the net synthesis of organic compounds used in cellular metabolism [3]. During oxidative phosphorylation, electrons are transferred from electron donors to electron acceptors such as oxygen, in redox reactions. These redox reactions release energy, which is used to form ATP. The oxidative phosphorylation-related Cytochrome c Oxidase function (KEGG term K02256) was actively expressed during the initial acclimation days reaching a peak at 72 h, declining rapidly soon after to levels similar to those at the beginning of the aquarium experiment (Figure 3B). The oxidative phosphorylation distribution profile was similar to that of methane metabolism (K08684), supporting a direct energy metabolism reliance between *B. azoricus* gills and methanotroph endosymbionts as shown in Figure 3B.

The lowest Cytochrome c Oxidase expression towards the end of acclimatization suggests a progressive decline in the capacity of *B. azoricus* general capacity to maintain its energy balance.

#### 3.3.2. Glycolysis-gluconeogenesis

Glycolysis is the process of converting glucose into pyruvate and generating small amounts of ATP and NADH [28]. Gluconeogenesis is a synthesis pathway of glucose from non-carbohydrate precursors. As a general tissue metabolism indicator, glycolysis in deep sea vent mussels may reflect the animal's metabolic fitness and its capacity to sustain the lack of external energy sources when maintained in aquaria. Surprisingly, the levels of gluconeogenesis transcripts (KEGG term K01596) remain fairly constant from the beginning to the end of acclimatization, suggesting basal sugar metabolism preservation in aquaria conditions (Figure 3B).

#### 3.3.3. Protein kinase

Protein kinases are key regulator enzymes of cell function, constituting one of the largest and most functionally diverse gene families. They modify other proteins through phosphorylation, inducing functional changes of the target proteins, directing their activity, localization and overall function, thus coordinating the activity of almost all cellular processes [29]. Kinases are particularly conspicuous in cellular pathways acting as regulators of the cell cycle and signal transduction. In the present study, levels of the protein kinase KEGG function K08884 were at basal levels, without major changes throughout the acclimatization experiment. As was the case with Glycolysis-gluconeogenesis, the protein kinase transcript expression profile suggests a basal response to aquaria acclimatization, which does not seem to be directly affected by environmental conditions, nor changes in microbial diversity, but seems to rely on intrinsic mussel capabilities to endure long-term acclimatization (Figure 3B).

#### 3.3.4. Co-factors and vitamins

Co-factors and vitamins are organic compounds that are essential in trace amounts for the maintenance of normal metabolism and proper enzyme activity in fish and shellfish animals. They generally cannot be synthesized at adequate levels by the body and must be obtained from diet [30]. Ion cofactors are essential components of metalloproteins that are important for a variety of cellular functions such as storage and transport of proteins, enzymes and signal transduction proteins[31]; they have also been implicated in infectious diseases. The metabolism of cofactors and vitamins is here represented by the transcript assignment Ferritin (to KEGG term K00522), a key functional protein involved in iron homeostasis and oxidative stress response. It was expressed starting at 12 h of acclimatization and increased over acclimatization time (Figure 3B). As previously demonstrated, Ferritins are significantly regulated by the symbiont content of vent mussels, driving host-pathogen, as well as host-symbiont interactions [32]. An increase of this functional transcript from the beginning of week 2 is indicative of important metabolic adjustments to aquarium conditions. This is most likely related to oxidative stress induced by environmental factors, loss of endosymbionts from gill tissues, and ion metal imbalance and metabolic shifts, causing Ferritin transcription to increase and regulate Fe storage within *B. azoricus* gill tissues.

### 3.4. Genetic information processing

Genetic information flows from DNA into protein. This flow of information occurs through the sequential processes of DNA transcription to RNA and translation from RNA to proteins. Transcript abundancies during acclimatization in four functional categories related to genetic information processing were measured (Figure 3B) and are discussed below.

#### 3.4.1. Transcriptional machinery

Regulation of transcription requires transcription factors in both prokaryotes and eukaryotes. These factors are responsible for stabilizing binding interactions and opening the DNA helix to allow the RNA polymerase to access the template. Representatives of this functional category (KEGG term K05692) are observed in increasing percentages from week 1 of acclimatization, reaching their highest expression at the end of experimental acclimatization. This pattern is consistent with the physiological reactions of vent mussels to aquarium conditions, with increasing activity of the transcriptional machinery likely resulting in higher protein synthesis.

#### 3.4.2. Spliceosome

The spliceosome catalyzes a reaction that results in the intron removal from the primary transcript mRNA, joining together the protein-coding exons in a process generally referred to as “RNA splicing”. Only Eukaryotes have spliceosomes. Spliceosomes form large complexes assembled around proteins and RNA on the newly synthesized precursor messenger RNA, and recognize splicing sites between introns and exons. The spliceosome functional category (KEGG term K03283) showed a similar pattern of expression as “transcription” (section 3.4.1. above), increasing after one week of acclimatization and reaching its highest level after five weeks. This expression is consistent with an increase in transcriptional activity towards the end of experimental acclimatization.

#### 3.4.3. Translation

Translation related transcripts were represented by the category functional “translation factors”, KEGG terms K03231 and K03234. Their level of expression showed a slight increase by the end of week 1, denoting the general pattern of expression also seen in the preceding genetic information processing stages (see sections 3.4.1. and 3.4.2. above).

#### 3.4.4. Ribosomes

Ribosome-related functional categories (KEGG K02981 and K02975) were the largest represented categories, corresponding to 19.50% of all the identified transcripts at the end of acclimatization. Starting at 24 h after the beginning of acclimatization their expression increased over time, reaching highest transcript levels after 5 weeks. Being the most represented of all functional category assignments, they may be regarded as a good indicator of the transcriptional status of vent mussels in aquaria during the entire experimental acclimatization period. Ribosome-related functional categories provide evidence for major transcriptional changes during adaptation to new environmental conditions in which protein synthesis-related transcripts assume preponderance over other categories of transcripts.

### 3.5. Replication and repair

DNA replication and repair processes identify and correct DNA damage that occurs within the cells during normal metabolic activities and under the influence of environmental factors. Structural damage to the DNA molecule incurring from environmental factors may alter or impair the cell's ability to transcribe the genes affected by the encoding DNA. This functional category transcript was represented by the KEGG assignment K11251, “Histone and exomal protein” and showed an increased pattern of expression toward the end of acclimatization, consistent with possible DNA damage accumulating over time, when vent mussels are acclimatized to aquaria conditions.

### 3.6. Degradation-chaperones and folding catalysts

Molecular chaperones are well known protein facilitators of other protein folding and assembly and are ubiquitously present in cells, coping with stress-induced degradation and environmental factors [33]. Heat shock proteins (HSP) are a family of proteins that are produced by cells in response to exposure to stressful conditions. Many members of this group perform chaperone function by stabilizing new proteins to ensure correct folding or by helping to refold proteins that were damaged by cell stress [34],[35]. HSPs bind to misfolded cellular proteins, facilitate proper folding, and prevent protein aggregation. Degradation-chaperones and folding catalysts functional category (KEGG term K04077) was expressed at higher levels within the first hours of acclimatization, progressively decreasing over 5 weeks' time (Figure 3B). This is consistent with levels of DNA strand breakage and HSP70 expression in response to decompression stress during the first 36 hours of adaptation to sea-level pressure [36]. A lower level of HSP over 5 weeks' time is suggestive of a progressive state of normalcy as far as RNA degradation and protein folding are concerned.

### 3.7. Transport and catabolism-phagosome activity

Phagosomes are phagocytic vesicles in which microorganisms can be killed and digested. They are an integral part of the phagocytosis process, an important cellular immune reaction to destroy microbes. Phagocytosis is central to tissue remodeling, inflammation, and defense against infectious agents and one of the most important defensive functions of hemocytes in bivalves, including *B. azoricus* [37]–[39]. A phagosome-tubulin KEGG pathway (K07374, K07375) was found to remain unaltered until 48 h, after which time levels of expression started increasing, reaching highest levels toward the end of acclimatization. During phagocytosis, cytoskeleton remodeling involves the participation of microtubules composed of tubulin subunits and other phagosomal proteins. Interestingly, an increase of phagosome-related transcripts appeared to be concomitant with the expansion of gill microbial diversity around 3–4 weeks of acclimatization pointing to an increase of phagocytosis activity in response to opportunistic bacteria prevalent at the end of acclimatization.

## 4. Conclusions

A metatranscriptomic study was carried out to analyze *B. azoricus* gill-microbe associations during an acclimatization experiment conducted in plain sea water aquarium environment and at atmospheric pressure. rRNA sequencing analyses from 11 transcriptomic data sets, corresponding to distinct acclimatization time points, highlighted a variable distribution of taxonomical and functional assignments, consistent with changes in symbiont metabolic activity, as vent mussels were progressively acclimatized to aquarium environmental conditions. Between 1 week and 2 weeks a drastic change in OTUs distribution was detected, pointing to a clear reduction of *Oceanospirillales*, *Methylococcales* and Thiotrichales which may have disappeared after 4 weeks of acclimatization. From 3 weeks onwards an increase of bacterial diversity was seen, mostly represented by Campylobacterales.

An increase of eukaryote species corresponding to Bivalvia and Bryozoa was detected from 2 weeks onwards, representing almost all taxonomy assignments by the end of acclimatization.

Functional assignments revealed variable expression across time points and distinct metabolic pathways shifts. Methane metabolism activity was markedly influenced by aquarium conditions, showing sharp decline after 72 h. This microbial composition variation is supported by evidence suggesting opportunistic bacterial colonization of host gills after 2 weeks of acclimatization (Figure 2). Several reasons may explain microbial community alterations during vent mussel long term maintenance in aquaria. The loss of endosymbionts from the beginning of week 1 is detrimental to vent mussel capacity to survive outside its natural environment, shaping the animal's capacity to utilize external sources of energy, influencing its transcriptional activity and altering its capacity to overcome external encounters with environmental microbes in aquaria (Figure 2).

The analysis of transcript functional category patterns during 5 weeks of experimental acclimatization revealed that several well-known functional systems of genes and signaling pathways, associated with stress responses and energy metabolism, were qualitatively altered, highlighting 3 major subsystems trends: (i) increase of “genetic information processing” transcript levels toward the end of acclimatization; (ii) decrease of “chaperone and folding catalysts” and “oxidative phosphorylation” transcripts toward the end of acclimatization; (iii) relatively unaltered “gluconeogenesis and co-factors-vitamins” related transcripts.

To the best of our knowledge, this *B. azoricus* endosymbiont-host metatranscriptomic analysis has provided, for the first time, a qualitative analysis into microbial diversity and gene expression patterns, demonstrating the gradual loss of gill endosymbionts, under sea-surface-level environmental conditions.
